# Sampling of environmental electromagnetic frequencies demonstrates the evolution of the nervous system toward social cognitive reflexes

**DOI:** 10.3389/fncom.2023.1008238

**Published:** 2023-02-23

**Authors:** Joseph A. Hazani

**Affiliations:** United States Gypsum, Los Angeles, CA, United States

**Keywords:** sensory perception and regulation, sampling theory, signals and systems analysis, social evolutionary theory, frequency domain analysis (FDA)

## Abstract

The aim of this research is to help inspect the motion of cell life by applying electrical engineering scientific techniques to the cellular evolution of human neural networks. Using a mathematically rigorous theory of cellular biological progression, the hypothesis will demonstrate that cell life evolves toward increasing the organism's resonant energy transfer or “exposing points” with its natural environment. This increases the sampling points of electromagnetic radiation frequencies available for transferal into its biological system to stabilize its cellular reproduction cycle as a function of physical motion measurable in volt-ampere or calories/second as an experimental measure of fitness.

## 1. Introduction

The aim of this research is to help inspect the motion of cell life by applying electrical engineering scientific techniques to the cellular evolution of human neural networks. The hypothesis will demonstrate that cell life evolves toward increasing the organism's resonant energy transfer or “exposing points” with its Natural environment ergo the sampling of electromagnetic radiation frequencies available for transfer into its biological system. The proposed theory claims the stability of its cellular reproduction cycle as a function of physical motion transfer is measurable in volt-ampere or calories/second.

The cellular control of electromagnetic radiation transmission is diagrammable by applying linear time-invariant (LTI) systems theory and digital signal processing mathematical principles. Indeed, introducing LTI systems modeling to cellular biology is principled on the experimental evidence of piezoelectricity and electron resonance transfer as a natural phenomenon (Shamo and Lavine, 1967):

Resonance scattering of electrons is probably one of the most remarkable and fundamental phenomena known in electron diffraction…Effects associated with resonance scattering are frequently observed in atomic, molecular, and nuclear collisions (Peng et al., 2004).

It follows that an electronics paradigm for evolutionary biology can be introduced for further understanding of human cellular networking and its objective to stabilize reproductive growth. Indeed, there is evidence of neural oscillations experimentally demonstrated in the neurobiological literature (Milton et al., 1990; Fröhlich and McCormick, 2010).

There is copious evidence of electrical phenomena in cellular organisms, with successes in electrical circuit analog models of cellular nervous system responses, for example, the Hodgkin–Huxley model is one classic example (Hodgkin and Huxley, 1952). It is proposed that the electrical feedback neurocircuitry of cellular organisms can be inspected using linear time-invariant control theory principles, for example, bounded-input bounded-output (BIBO) stability criteria. These principles give us quantitative insight into the time evolution of cellular phenomena into higher electrical feedback orders of controlled or organized movement in the natural environment, i.e., genetic fitness. It is claimed that this includes a continuum of inter-human neural cellular feedback networks in societies that promotes human progress in conceptual language as measured by the advances in mathematical and natural scientific literature over the last several hundred years. These advances have corresponded with the stable amplification of *de novo* calorie transfers, i.e., commercial trade activity, generating an exponential growth in the human population genetics, demonstrating the genetical fitness of the human race.

## 2. Information and the organism

We must familiarize ourselves with the current understanding of cellular evolution before attempting to understand human calorie productivity as principled by cell life. Let us first introduce the irreducible mechanisms of cellular replication: *replication, transcription*, and *translation* of the cell. The molecule deoxyribonucleic acid (DNA) produces copies of itself, which are then transcribed by ribonucleic acid, which is then passed onward to translate the copy into proteins, the building blocks of cellular signaling. The phrase *Central Dogma* coined by Francis Crick encapsulates the vitality of this process. It is a process that occurs within every cellular organism on Earth. Here, as advanced by biological scientific knowledge, is where species of life on Planet Earth originate. Within this iterative process, changes in cellular reproduction arise *vis-à-vis* previous cellular generations.

Known commonly as “mutations,” very rarely do they become pronounced enough to have any lasting effect on the organism. Most mutations are catastrophic at promoting stable cellular reproduction cycles, providing physical evidence of the meticulousness of cell life in preventing replication “errors” during cellular divisions. Yet “errors,” or interpretively the “modulation” of nucleic acid sequences between cellular reproduction generations, *still* occur as a natural phenomenon of cell life. Organisms that have experienced genetic mutations, and have successfully reproduced or self-replicated over sufficient generations, continue to propagate those changed nucleic acid indices within their cellular engine until the protein expressions of the organism are modified and physically form the attributes of the organism's cellular reproduction stability, i.e., *traits*. It is the continual expression of these stable mutations which creates divergences or *variations* within organisms in the biological domain. It is these divergences that Charles Darwin observed to be a process he popularized as “Natural Selection,” contrasting it from the human-handed selection of domesticating wildlife (dogs, plants, livestock, etc.).

According to the Natural Selection paradigm, stable mutations do not necessarily *cause* the organism whose genetic data are modified to reproduce or *iterate* it, furthering its heredity. Rather, species and the features of species that promote the regularity of cellular reproduction (i.e., *adaptations*) emerge within an organism's population *via* a hypothetical *stochastic signaling process* between cellular generations in continuous contact with the cellular environment. The physical demonstration of cellular “fitness” is a healthy mutation that causes the increased stability of one organism's genomic iteration within a species inter-generationally, as measured by the sampling rate.

Yet within this overview, we have glanced over an obvious and glaring fact. Reviewing the Central Dogma once again, the process observed is intimately related to an anthropic concept of “information.” It is hypothesized that this machinery, then, is ultimately a *signaling system* and can be mathematically modeled using electrical engineering scientific techniques successfully applied and progressed to electron motion control, for example, electronics. Indeed, Hubert Yockey has already demonstrated the hypothetical modeling of the Central Dogma phenomenon as a digital communication channel in his work *Information Theory, Evolution, and the Origin of Life*.

Intuitively, if we consider the random walk, contingency-laden, model of evolutionary “progress”—that there is no scientific predictability to the Natural Selection of fitness and thus evolution is necessarily unpredictable and not possible to quantify—we stumble upon a blatant paradox when considering the observation of biological complexity on Earth. Because one of the defining features of an organism is its stable cellular replication, *metabolic* maintenance, i.e., physico-chemical energy measurable in *calories*, is required to sustain its mobilization or locomotion of cyclical repetition, for example, hormonal production. Cell tractability or movement, for instance, directed at the aims of persisting the *stability* of its cell life through sampling *ergo* transferring *ex vivo* energy—namely *natural quantities of thermal electromagnetic radiation*—in its environment already demonstrates a self-evident contradiction to the random walk argument proposed by notable evolutionary biologists such as Stephen Jay Gould. Logically, cellular motion, which requires a *metabolic exercise* of thermal energy to become locomotive, i.e., *physical work*, is necessarily *stabilizing* to cellular reproduction because it creates or *gains* greater limits of transferable energy potential for the cell in its current physical position in its environment. In other words, the additional calorie output of cellular locomotion relational to a static position or the absence of motion, while causing the depletion of the kinetic energy of the cell, hypothetically *improves* its cellular fitness through the greater possibilities of re-energizing the cell's reproduction through the resonant energy transmission phenomena, like piezoelectricity and the crystal oscillation discoveries (now embedded in microelectronics) in the twentieth century.

The greater possibilities of calorie transmission *per cellular reproduction cycle* necessarily increase calorie potential equivalently measurable in the electromagnetic scientific domain in voltages. Hence, *mobility* in the cellular environment is a considerable adaptation in producing stable regenerative feedback. The cellular evolutionary record is testimony to this: Nature selects for adaptations that increase the *rate* of *sampling* electromagnetic energy from the environment into the closed-loop cellular control system or *organism*, providing the organism with greater thermal efficiencies for energy transfer, necessarily creating energy available for physically sustaining its reproduction cycling and therefore fitness.

## 3. Biological society as multi-organism feedback control system

Having now proposed the beginnings of synthesizing the applied mathematical scientific techniques of electrical engineering to evolutionary cellular theory, this mathematical hypothesis will be synthesized with the cellular phenomenon of the *society* to further elucidate the evolutionary and social neuroscientific implications.

Human society, even at its most fundamental organizing principle of cellular reproduction—male and female—is a form of biological adaptation. It can now be made clearer why this is true, not just for humans, but for all social organisms: societies expand the quantitative rate of the sampling and transfer of environmental or *ex vivo* electromagnetic radiation by cellular organisms, both within and external to the group population. This explains the phenomenal evidence of the development of social neuro-cellular traits, e.g., language, in humans—sexual dimorphism included—as a qualitative measure of cellular tissue differentiation complexity.

**Figure 1:**
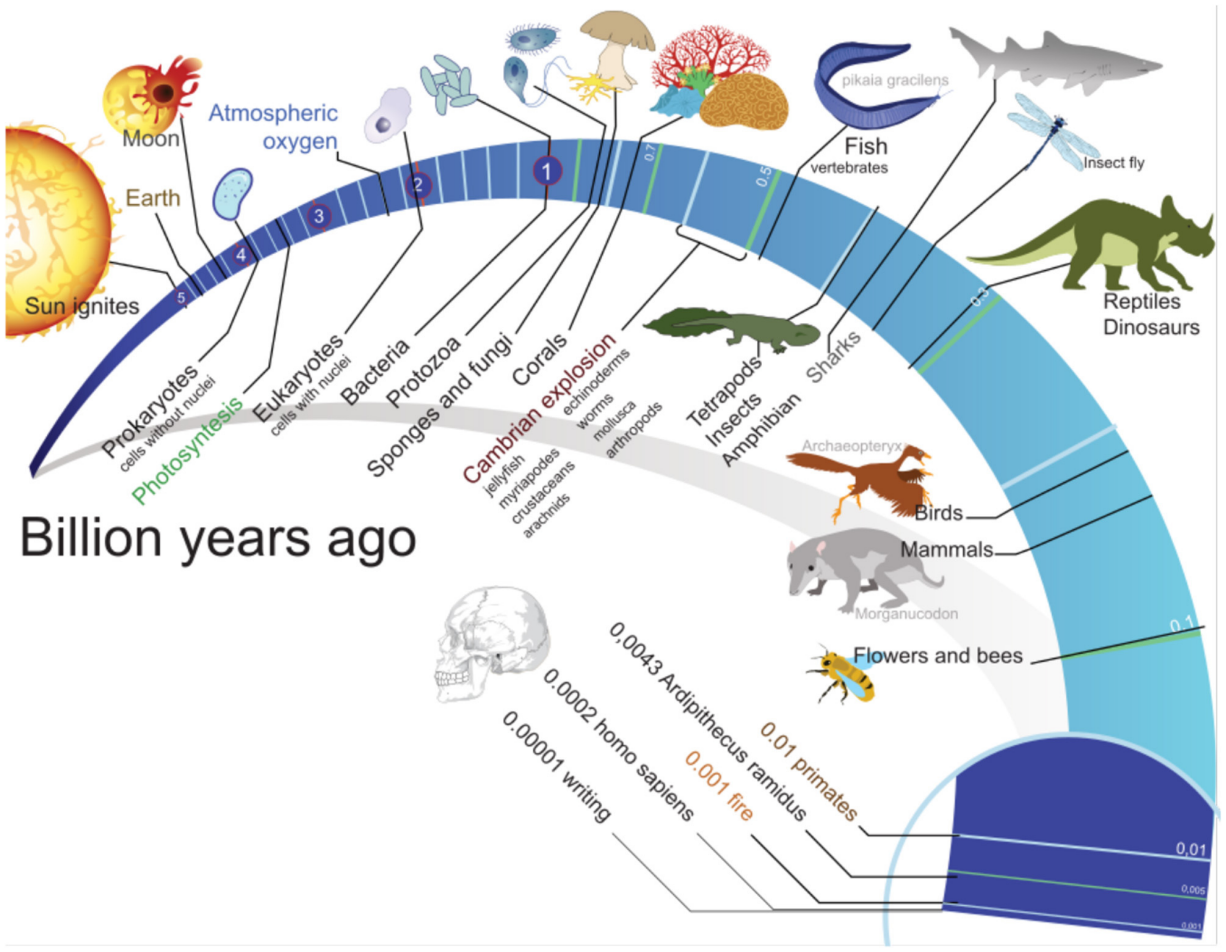
Timeline of evolution of life (2012). Mariana Ruiz Villarreal (User: LadyofHats/Wikimedia Commons).

It is proposed that greater quantities of stable transmission of radiation between cells in their environments cause an increase in the boundaries of signal processing—*bandwidth*—in the society *ergo* greater calorie feedback gain or thermal regenerative feedback with the external environment, and therefore demonstrates improved cellular fitness in relation to the prokaryotic cellular domain. Societies, therefore, cause gains to the sampling feedback rate or electronic impulse control responses of cellular organisms measured in electromagnetic radiation sampling cycles in proportion to gamete cellular division cycles through extending a faster feedback response—larger *Nyquist sampling rate*—of the thermal radiation organism's environment. This greater quantity of radiation transmission into the multi-organism neural feedback network necessarily expands the mechanical limits of electronic oscillations controllable or electric potential available for causing action potentials in the biological group, thus extending the boundaries of thermally stabilizing the group's reproduction fitness. This includes adaptations for controlling environmental noise, i.e., *information entropy*. This hypothesis leads to the judgment that the observation of the progression of cellular complexity in Earth's fossil records to the human cerebral cortex (see Figure 1) is *geometrically* *proportional to sampling speeds or quantities of information gain* measured by calories per circadian cycle. It is proposed that an electromechanical LTI block diagramming of cellular organism radiation transfer can numerically order cell life in complexity according to the organism's countable feedback control elements or loops to demonstrate cellular evolutionary progress toward reflexive nervous systems which can sustain greater quantities of electronic oscillations per second for sampling the environment, for example, the human brain.

Returning to the understanding of social animals and how “socializing” is an adaptation, multi-cellular organism communication using *mutual* social cognitive reflexes theoretically causes greater self-certainty of quantities of radiation transmission or *quanta* in the society as an effect of the greater feedback rates imposed on each of the individual organisms through the inter-generational development of biological group traits which improve harmonizing radiation transmission between themselves socially. Logically, greater quantities of quanta transmitted within biological societies per unit time expand the quantitative boundaries of an action potential, thereby increasing the bandwidth of electromagnetic radiation processing in achieving homeostasis per gamete reproductive cycle. Hence, it is biologically necessary that the genetic signaling that is contributory to this multi-cellular system stability or group fitness will be transmitted in a hereditary fashion, i.e., *recursively*, thus evolving group selective traits in particular cellular organism's reproduction cycles in time. This logically will occur in brain cellular tissue as a process of achieving homeostasis. Human beings self-evidently demonstrate the greatest signal processing rates in cell life.

It is a digression to characterize *what* kind of radiation is transmitted and expressed phenotypically, i.e., the genetic material and physical protein expressions caused by group adaptations. Because of the innumerability and the impenetrable mysteries still residing in the biological kingdom, for example, the unknown origin of cellular life, generalizing is poor practice. Yet several examples can help the reader better understand the recursive structuring of cellular organisms in causing faster sampling rates or responses (societies) as correspondent to the biological scientific theory being established.

**Figure 2:**
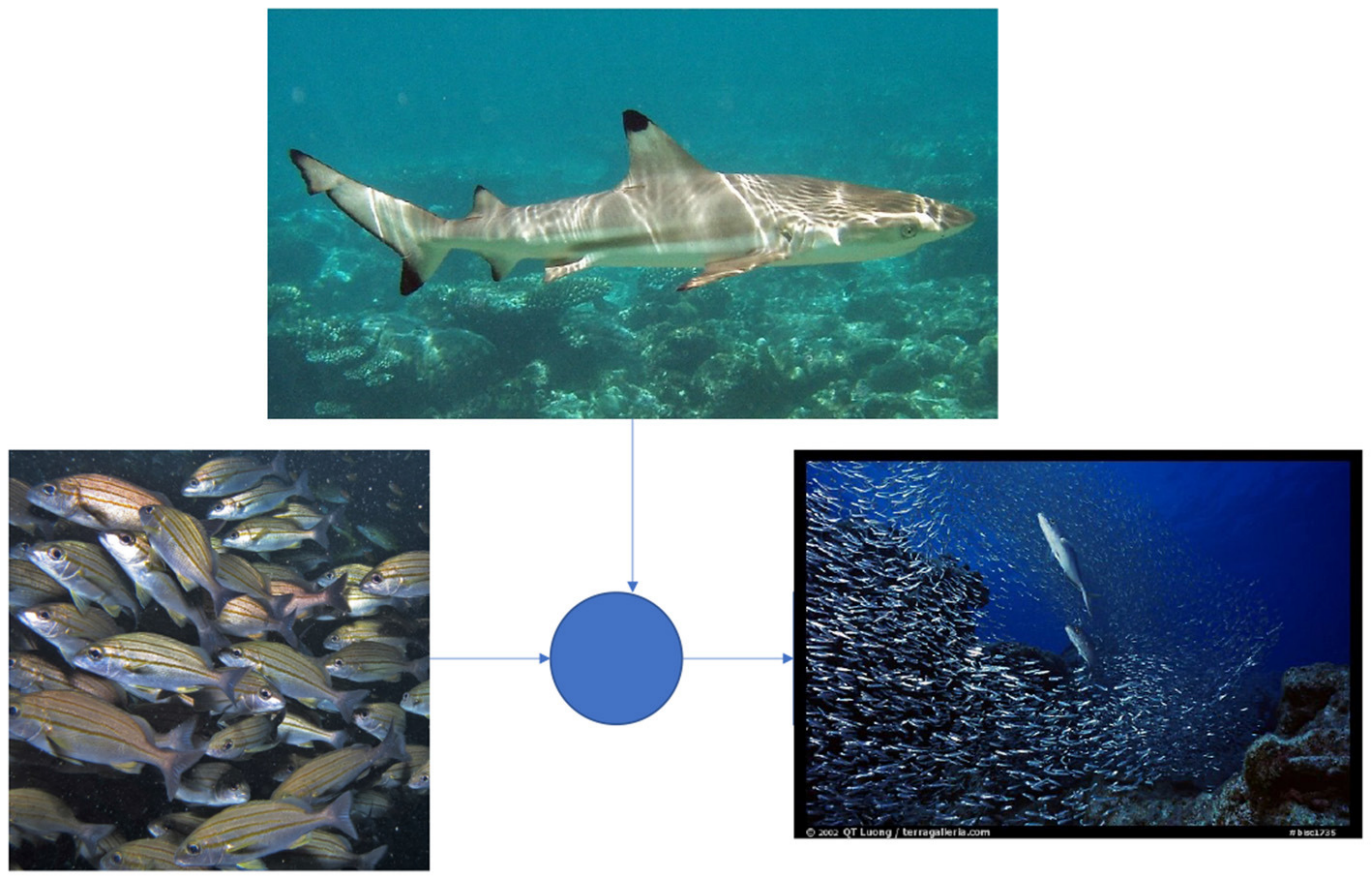
Feedback representation of a school of fish depicted as a cellular network of nervous tissue responding to predatory environmental disturbance to their mutual stability of cellular replication (Striped Grunters © Derek Keats All Rights Reserved. Blacktip Reef Shark by fishx6).

For instance, we can consider the advantage of societies to repel predators more effectively through their neuromuscular coordination which hypothetically forms *natural* communication channels or continuums, i.e., “analog filters,” of radiation transmission detected by their sensory stems in relation to the external forces outside of their society (see Figure 2). “Predator evasion” may not directly be evident as a qualitative measure of system stability but let us consider it for now.

If we diagram the predator to be a discrete quantity of harmful radiation (“disturbance”) external to the continuum of organism replication of the society, then it is logical that the phenomenon of the instantaneous coordinating of individual organisms in the group of a social species—say a school of fish or a flock of birds—promotes cell life and will promote the environmental feedback ordering in and multi-organism toward, say, nervous reflexes which are conditionally activated according to one organism in the population physically reacting to the adrenal feedback responses of another. For instance, the nervous system in fish is very selective:

The olfactory system in vertebrates comprises a highly specialized sensory organ for detection and identification of minute quantities of chemicals in the environment. Experimental studies have documented the role played by olfactory information in social relationships, prey or predator recognition, and the search for food (Døving, 1986).

Such environmental feedback response rates can be mathematically analyzed to understand the causes of an extension of the electromagnetic frequency continuum—or *bandwidth*—within the entire population of the society improves sampling quantities per unit time or sampling rates of predatory movement in the environment. Thus, the sampling rate of the radiation in the environment is necessarily faster within the biological group population than in a single cell or organism itself. This causes the population more stable calorie regeneration with the observable closed system thermal environment.

In other words, there is a quantitatively necessary and geometrical, i.e., mechanical, limitation to the stable feedback rate or *channel capacity* of the quantities of radiation transmittable measurable in spectral irradiance. Yet, while developing adaptations of social cooperation through unified recursive feedback mechanisms or *group biological traits*, the higher limits of stable quantities of communication of electromagnetic radiation such as heat by the society in its radiation environment will lead to statistically significantly higher rates of cellular reproduction survival in the population.

We can, therefore, diagram the penetration of a shark into a school of fish in terms of thermal disturbances piezoelectrically activating the fish society's nervous system synaptic integration to diagram the society as a complete cellular body comprised of divisible cell life. Through the electromagnetic flux conducted by the physical motion of the shark, the sensory perceptions of the fish are necessarily affected, such that fish with greater continuity of neural feedback responsiveness with other fish cause immediate polarization events in the nerve tissue. This is diagrammable in the LTI system theory paradigm, where group fitness can be observed as a phenomenon of neural tissue replication stability sequenced and therefore indexed inter-generationally, causing increases in social traits which increase the communication *spectrum* of the cellular population forming an integrated continuum of energy transfer with the society's environment. Societies and the nature of biological adaptations that have emerged to *stabilize* cellular reproduction cycles signify the importance of social coordination or communication reflexes as a measure of sampling rates of heat conductivity in cellular evolution.

This hypothesizes that social traits are not sufficiently molecularly genetical, i.e., *physically material*. Indeed, societies can, through the means of their inter-generational (recursive) processing of environmental radiation, *gain* genetic traits which are not paradigmatically “genomic” in cellular evolutionary theory. Human literacy is a prominent example. These genetical traits, such as the discovery and inter-generational transmission of the learning of the opening of a coconut by primates, for example, cannot be found molecularly in DNA, yet is necessarily a *hereditary* adaptation that improves the organism's fitness and therefore promotes heat conductivity rates by the cellular diagrammable society. The fitness is first promoted by the original individual member in the society to bear the discovery of the new calorie source in the absolutely summable bounded radiation environment the group is adapting to, and consequently, *signals* to the rest of the members of the population *de novo* gesticular control of the physical transfer of calories into the body through instrumentation, promoting the cellular replication stability of the biological group through the increased *memory* of calories in the population's *ex vivo* cellular environment feedback channel.

The more regular, i.e., stable, calorie exchanges between the primates^1^ and their habitats in time, compared to further time-dependent calorie uncertainties (entropy) of foraging, improves the radiation processing speed or *calculations rate* of the biological group due to the theoretically extended electromagnetic continuum between the cells and the *ex vivo* radiation self-certain boundary parameters of knowable heat capable of being used for population growth or *thermal region of convergence* as the society reproduces itself. This increased stability in society is demonstrative of the utilization of *natural electromagnetic resonances* in cellular evolution. The ability for a society to cause memory between sequences or generations of replication is possible by the total harmonics driven inter-generationally between each generational sequence of the society. We can, therefore, reason the complete predetermination of the natural progression of group traits of transmitting sampled quantities of thermal radiation or discrete-time radiation data using the mathematical techniques known to those in the electrical engineering sciences.

The hypothesis of radiation transmission in the presented paradigm through the natural electromagnetic frequency continuum is elegantly modular and succinct in providing a coherent representation of the natural phenomenon of neural feedback in cellular organisms. The signaling of organisms in societies can be mathematically diagrammed as particular cellular bodies adapting to their environment, including their surrounding biological group population; or a society as a cellular body itself adapting or responding to its *ex vivo* radiation environment; given the mathematical linearity of the underlying physical principles of resonance. Before advancing the hypothesis any further, however, it is necessary to examine the highest ordered radiation signal processor in human cellular life. And that is in the mathematically scientific examination of the highest ordered, i.e., *fastest*, signal processing of the human body, i.e., *consciousness*.

## 4. Human homeostasis

**Figure 3:**
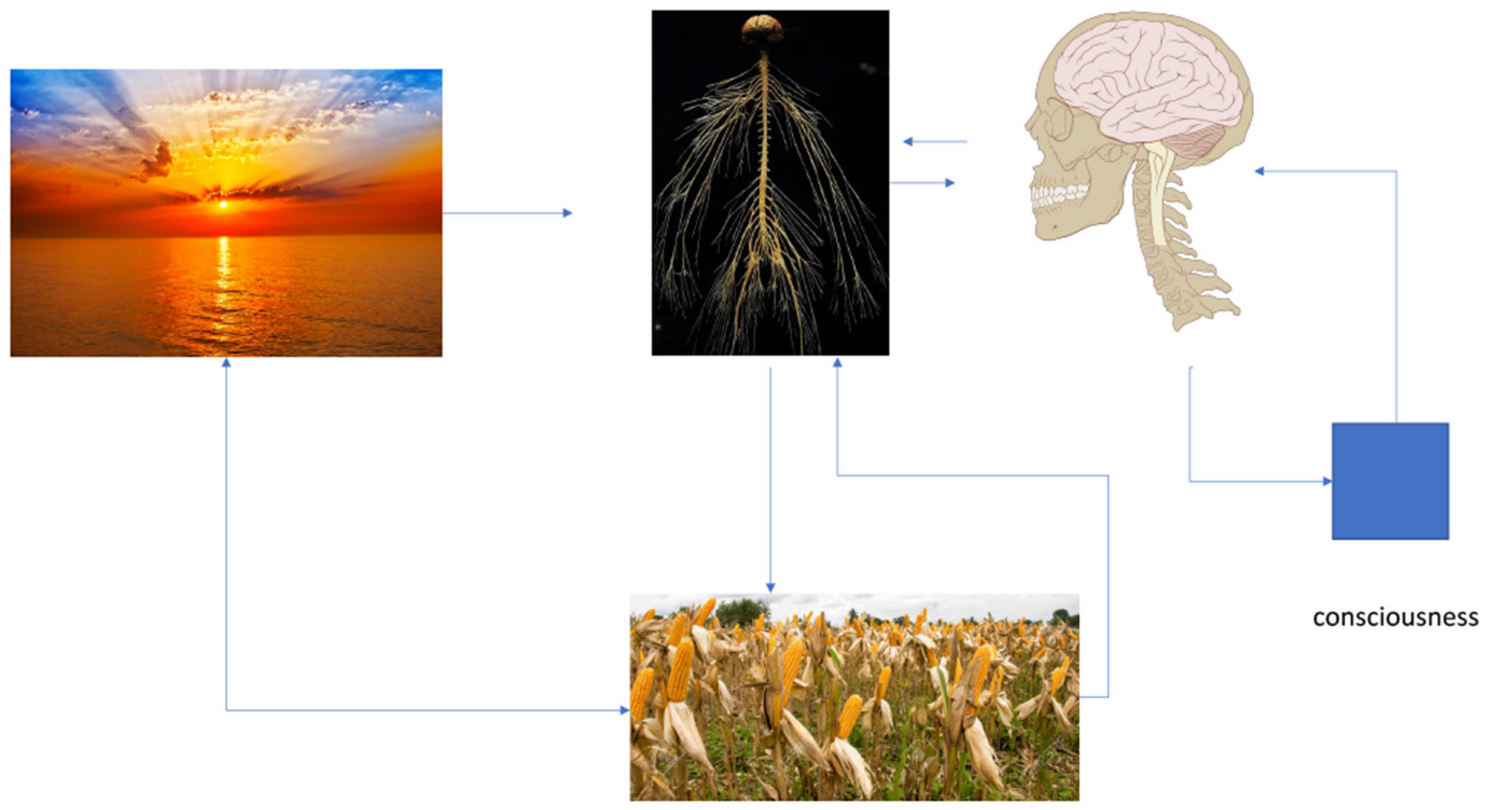
Natural concepts are used to illustrate the mathematical feedback diagramming of the electric signaling activity of self-consciousness by individuals on their human bodies as a necessary effect of stabilizing cellular reproduction through calorie transfers (such as corn) with the physical environment.

Once we hypothetically understand human society changes as the phenomenon of higher-ordered electromagnetic feedback networks between networks, we can now be more exacting in the mathematical diagramming. We can mathematically diagram human nerve cells as electronic feedback control systems functioning to stabilize their heat transfer, i.e., adaptations, in their local thermal radiation regions of convergences (see Figure 3). We can further intuitively demonstrate the mechanics of a human population's uniform *subjective* reflexive control, thereby furthering a natural scientific paradigm of realizing the primary motor control of human societies: it is *consciousness* or the *mathematical* continuum of reflexive signaling activity caused by electronic oscillations freely predetermined in the body's neural tissue; primarily in the cerebral cortex.

**Figure 4:**
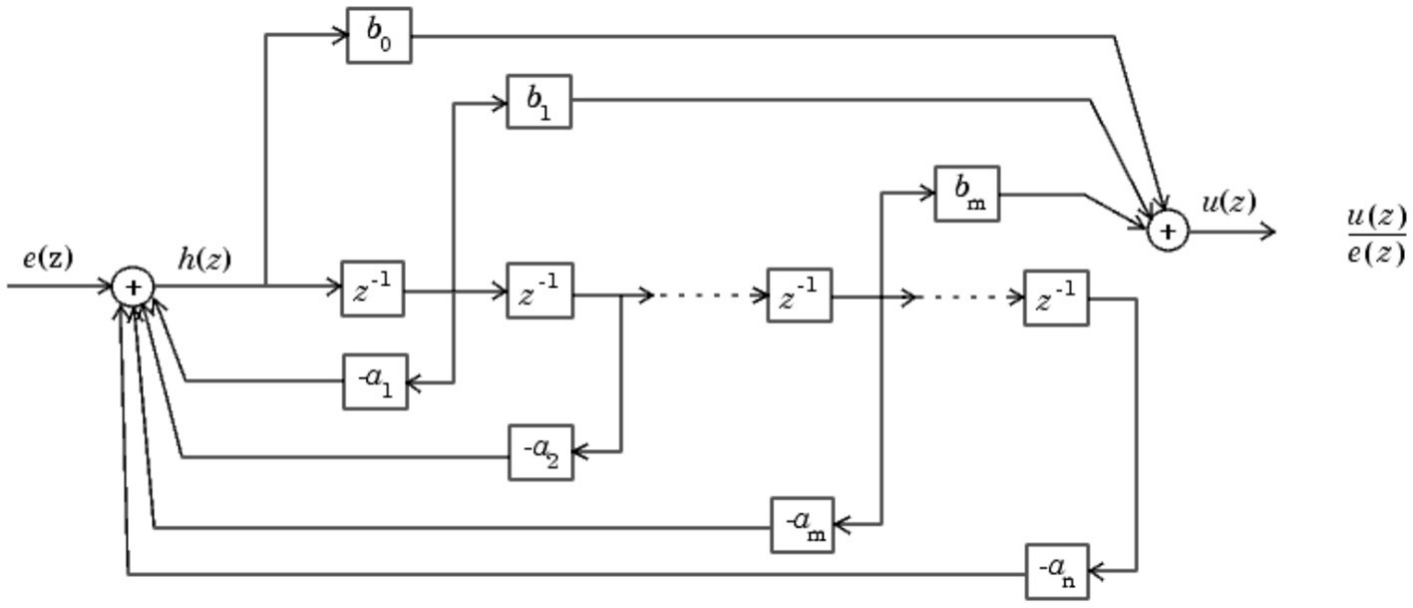
Example of a feedback realization diagram. Each control element counts as a feedback loop (MathWorks, 2022).

The neural reflexes freely causing human body physical movement can be mathematically realized as an LTI causal superposition of radiation signal sampling and processing with other human bodies, promoting stable harmonics of heat transfer or *homeostasis* of the human being's cell life (see Figure 4). It is proposed that the feedback realization diagrams of the electronics of the nervous system (in particular neural oscillations) can be examined by frequency domain mathematical techniques, e.g., frequency responses or Z-plane analysis, of *any* cell body's system response to stabilizing causal time-invariant analog electromagnetic radiation inputs, for example, sensory perceptions, to model external environmental electromagnetic effects (e.g., heat flux) on cellular reproduction cycles. This mathematical analysis permits the neuroscientific understanding of cellular evolution toward greater reflexive complexity *ergo* the understanding of the evolution of human consciousness as a function of thermal stability.

Yet, we may *prima facie* fail to see how conscious reading, writing, and arithmetic are acts of stabilizing cellular reproduction, for there is no immediate awareness that one is strenuously attempting to cause homeostasis, for instance, when one in fact is exercising his or her calories through an undeniable task of nervous system feedback transmission with his or her *exclusively* subjective electromagnetic frequency domain contained in the human body. We will demonstrate, then, that consciousness is the highest ordered discrete-time signal processor of electromagnetism, measurable by its progression in electromagnetic quantization rates in bits/second of the synthesis of sensory synaptic integration inputs.

As mentioned, there are multitudes of regularities, i.e., feedback control systems, that are self-evidently outside of the processing bandwidth of consciousness which nevertheless contribute to the homeostasis of the body, such as the parasympathetic regulation of the heart or the gastrointestinal metabolic process. We are claiming with mathematical formality, however, that consciousness is the *primary* mechanism of human society's homeostasis; it is the *digital* operation—or continuum of electronically *calculated* sequences—of nervous reflexive feedback action that *initializes* the physical motion of the subjective human's cellular body tissue in coordination with other human conscious awareness (consciousness). This continuum of electromagnetic sampling by humans hypothetically causes the greatest quantities of radiation transmission rates *ergo* the highest natural electromagnetic frequency spectrum or bandwidth possible to be digitally reconstructed by the populations' brain cells. This sampling rate can be concretely measured by the rates of electromagnetic transmission in W per day with the proliferation of telecommunication devices.

The reflexes of the brain are mathematically realizable as *digital* signals processing quantized electromagnetic radiation feedback inputs to control the body and the human subject's self-certain motion, i.e., self-consciousness. Consciousness, therefore, is hypothetically diagrammable as a digital *convolution* of physical radiation samples *numerically reconstructed* with the human body's necessarily proprietary electronic neural feedback network constructed by the self.

The measurement of the quantities of radiation available for sampling is linearly proportional to the *channel capacity* of the signal processing of consciousness in the body *via* the Shannon–Hartley equation^2^:

$$\begin{array}{l} C=B{\text{log}}_{2}\left(1+S/N\right). \end{array}$$

where C is the maximum amount of information or self-certain quantities of radiant power that can be reconstructed in the electromagnetic frequency continuum (communication channel) generated in the brain's conscious reflexive activity between itself with noise present (such as stochastic resonances), its *self-reflective capacity*, measurable in unitless bits/s. This activity is indirectly observable using imaging techniques such as fMRI and is electromechanically diagrammable through its connection to muscular tissue and the experimental determination of the neural tissue stimulation caused by the knowing consciousness or *self-consciousness*, where *knowing* originates logically as a mathematical continuum of piezoelectrically sustained electronic oscillations between nerve cells in the brain. Indeed, piezoelectrical phenomena are found ubiquitously in the biological domain.

**Figure 5:**
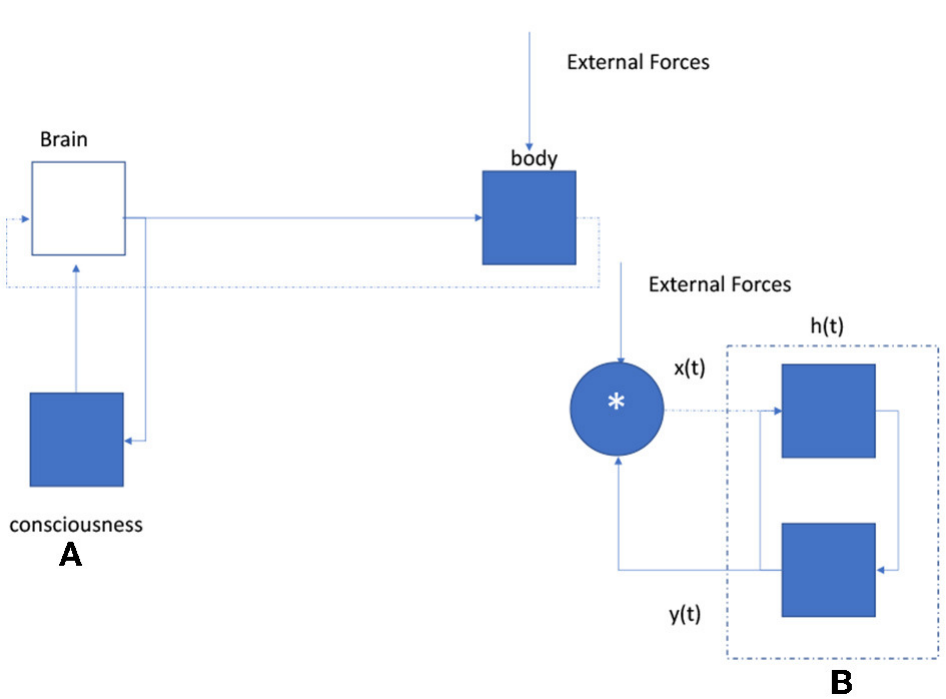
LTI diagram of conscious reflexive brain signals. **(A)** Consciousness is modeled as a digital signal processor hypothetically electromagnetically inducing continuous cognitive reflexes in the brain, causing the nervous system to create a mathematical continuum of recursive electrical reflexes in response to cellular environment (sensory) feedback inputs. The diagram hypothesizes that mental reflexes electrically caused by self-consciousness affect the body's movement. Higher bandwidths of sensory feedback inputs (e.g., the World Wide Web) cause a necessary expansion in the body's electric potential for electrically signaling, measurable in volt-ampere (V-A). This measurement can be converted to calories/second. **(B)** General LTI casual system response diagram of the physical feedback events creating the human body's cellular movement where H(s) is the transfer function. This mathematical realization conjectures a convolution operation of body feedback with new sensory inputs to cause a time series of conscious awareness data electromagnetically recorded in the brain for future reconstruction, i.e., memories.

Clearly, there are variegations of consciousness *fitness* or proprietary digitally constructed sampling filters causing differentials in measurable homeostasis in any observable human society and therefore in the radiant power capable of the human bodies in motion, measurable in V^*^A, and proportional to the *property* owned or *physically controlled* by the human being for sustaining its body or its families electric potential in the environment (see Figure 5). It is predictable, then, that human societies will evolve gradients in the exclusive conscious ability to extend calories, or *private property*, to other bodies. It is claimed that this phenomenon is observed in human societies as private property with contracts secured by lawful order.

## 5. Conclusion

In summary, it is hypothesized that nervous systems in the biological kingdom electromagnetically sample physical motion externally, and that cell life evolves toward increasing this rate of sampling, as demonstrated in the development of social genetical traits—and social cognitive reflexes—in cell life. It is proposed that these traits progress to such networking complexity that consciousness as cellular phenomena is generated digitally, i.e., a continuous formation of electronic neural oscillations causing numerical sequencing relations with its previous sensory feedback signal data known as the *self* . This self-certain necessary changing relation or calculation is the generation of the knowing self. It is proposed that free agency is necessary as an effect of permanent noise in the digital signal processing of the body.

A mathematically rigorous understanding of the networking effects of the electronics of human nerve tissue is possible due to the progress of the mathematical scientific techniques by the electrical engineering scientific tradition. Particularly, the ability to linearly electromechanically diagram nerve tissue reflexives as electronic feedback loops to analytically inspect for harmonic distortions or spurious frequencies with pure mathematical rigorousness.

**Figure 6:**
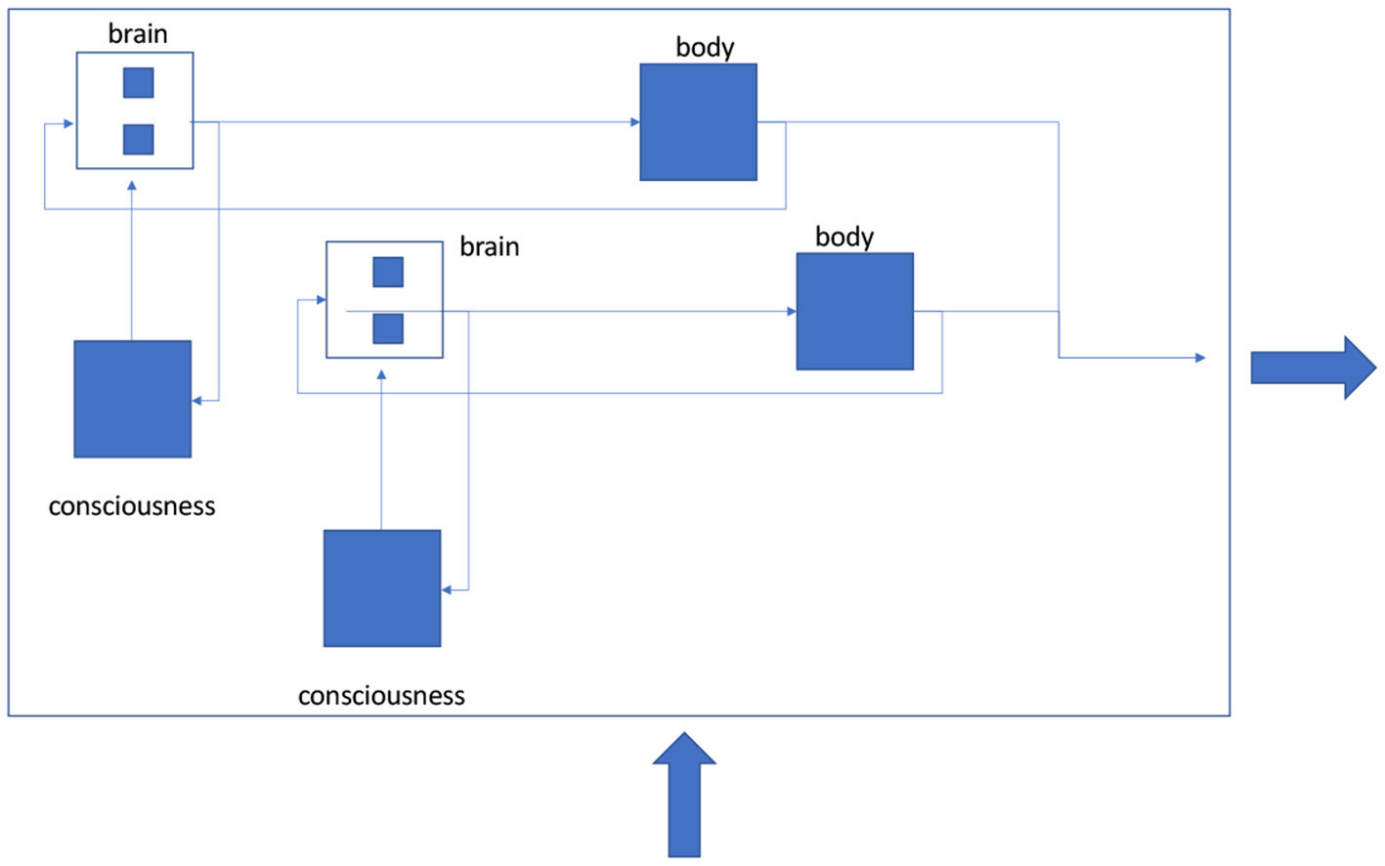
Conscious feedback operations of human cellular bodies in human society in response to universal external electromagnetic stimuli, for example, sunlight. The human society can be conjectured to be a linear superposition of self-conscious system responses to electrical reflexive activities. These responses are not necessarily universal or uniform. Indeed, “entropy” in the system creates opportunities for mutuality thus increasing the recursive structuring of the calorie exchanges between the cellular bodies in their environment, thereby promoting each body's cellular fitness or means for reproduction.

It is predicted that the more cellularly complex the organism, the more responsive it is to physical disturbances acting upon it, promoting its cellular reproduction ability or *homeostasis*. This responsiveness is measurable by the organism's sampling bandwidth of *ex vivo* electromagnetic frequencies (see Figure 6). This is physically demonstrated by the capitalizations in semiconductor and information technology over the last half-century globally.

It is proposed that neuroscientific disciplines introduce Signals and Systems Theory into their curriculum, such as *Linear Signals and Systems Second* Edition by B. P. Lathi; as well as digital signal processing theory, such as *Discrete-Time Signal Processing* International Edition by Oppenheim and Schafer; for the mathematically rigorous demonstration of the conscious feedback control mechanisms of human bodies to demonstrate original conscious reflexive activity as the human waking subject.

## Data availability statement

The original contributions presented in the study are included in the article/supplementary material, further inquiries can be directed to the corresponding author.

## Author contributions

The author confirms being the sole contributor of this work and has approved it for publication.

## Footnotes

^1^See: *Chimpanzees Sophisticated Use of Tools*, BBC Studios. https://www.youtube.com/watch?v=5Cp7_In7f88 (accessed April 4, 2008).

^2^Shannon's Theorem. Lehre. http://www.inf.fu-berlin.de/lehre/WS01/19548-U/shannon.html (Accessed 6 May 2022).

## References

[B1] Døving K. B. (1986). Functional properties of the fish olfactory system, in Progress in Sensory Physiology. Progress in Sensory Physiology, vol 6, eds. Autrum Ottoson D. Perl E. R. Schmidt R. F. Shimazu H. Willis W. D. (Berlin, Heidelberg: Springer).

[B2] Fröhlich F. McCormick D. A. (2010). Endogenous electric fields may guide neocortical network activity. Neuron. 67, 129–143. 10.1016/j.neuron.2010.06.005 20624597 PMC3139922

[B3] Hodgkin A. L. Huxley A. F. (1952). A quantitative description of membrane current and its application to conduction and excitation in nerve. J. Physiol. 117, 500. 10.1113/jphysiol.1952.sp004764 12991237 PMC1392413

[B4] MathWorks (2022). Direct Form II. Available online at: https://www.mathworks.com/help/fixedpoint/ug/direct-form-ii.html (accessed May 6, 2022).

[B5] Milton J. G. van der Heiden U. Longtin A. Mackey M. C. (1990). Complex dynamics and noise in simple neural networks with delayed mixed feedback. Biomed. Biochim. Acta. 49, 697–707. 1964553

[B6] Peng L. P. Dudarev S. L. Whelan M. J. (2004). High-Energy Electron Diffraction and Microscopy. New York, NY: Oxford Science Publications; Oxford University Press Inc. 186.

[B7] Shamo M. H. Lavine L. S. (1967). Piezoelectricity as a fundamental property of biological tissues. Nature. 213, 267–269. 10.1038/213267a0 6030604

